# A Case of Shoulder Presentation

**Published:** 1868-04-15

**Authors:** B. F. Ross

**Affiliations:** South Pass, Ill.


### A CASE OF SHOULDER PRESENTATION.

BY B. F. ROSS, M.D., SOUTH PASS, ILL.

[Noticing in the JOURNAL of the 1st inst., the "Report of a Case of Instrumental Delivery" in a case of Shoulder Presentation, in which the writer asks some "sage practitioner" to suggest a better method than amputation, I concluded to forward the notes of a somewhat similar case to you,—not claiming to be a sage amongst the members of the profession, however,—for your consideration and disposal.]

Mrs. H., aged 34, of a moderately strong constitution, and who had given birth to six children—the third child presenting the shoulder, and the physician in attendance amputated the arm at the shoulder—was again seized with labor pains about one o'clock A.M., April 2nd, 1865. There being no physician within five miles of her residence, a midwife was called in at three o'clock A.M.; the liquor amnii having been discharged about one hour before her arrival. Thinking that all things were doing well, she did not make an examination until the lapse or two or three hours, when, to her great astonishment, she found the hand protruding from the mouth of the womb. Not knowing what to do in this dilemma, she concluded to wait, hoping—as she expressed it—that something would turn up. The fates not favoring her, however, she concluded, at five P.M., to call for a physician. I was accordingly called at six o'clock. On making an examination,

I found the right shoulder presenting, with the head towards the right acetabulum, the right arm in the vagina close to the os externum, and grasped by the os uteri as firmly as though it had been placed in a vice, very much swollen and discolored. After repeated attempts to introduce my hand, I concluded that it was impossible to turn it without first placing the patient under the influence of chloroform, which I did not have, nor could get short of Jonesboro, some ten miles distant. On stating my opinions and conclusions to the friends, they insisted I should amputate, as Dr. P. had done in the previous case. This I could not consent to do. Owing to circumstances over which I had no control, I did not get the chloroform until the middle of the next day. I in the meantime had called to my assistance Dr. Dodds, of Anna, who administered the chloroform; and when perfectly under its influence, I again introduced my right hand, and finding it impossible to get my hand above the elbow of the child's arm without using more force than I thought prudent, I grasped the arm at the elbow as firmly as I could, and pushing steadily and firmly upwards and a little to the left, I soon had the satisfaction of feeling the arm ascend gradually into the womb, which I found perfectly relaxed. As soon as I found my hand in the cavity of the womb, I loosed my hold on the arm, and gliding my hand along the breast and abdomen of the child until I came in contact with the right leg, which I grasped by the knee, and completed the version by bringing down the foot, without waiting to hunt for the left foot. As soon as the patient had recovered from the influence of the anæsthetic, I gave her *Pulv. ergotæ*, ʒ ii, and in the course of fifteen or twenty minutes the womb had commenced to contract forcibly, and the child was delivered in about one hour. It may be urged that, inasmuch as I did not save the life of the child, I might as well have amputated the arm; but to this objection I would answer, that if I had been able to get the chloroform as soon as I could have reasonably expected, I think the result, so far as the child was concerned, would have been different. The mother made as good a recovery as could have been expected.

